# Psychotherapy for complex post-traumatic stress disorder: efficacy and therapeutic factors

**DOI:** 10.3389/fpsyg.2026.1684921

**Published:** 2026-02-09

**Authors:** Cenya Katalan, Human-Friedrich Unterrainer, Omar C. G. Gelo

**Affiliations:** 1 Faculty of Psychotherapy Science, Sigmund Freud University, Vienna, Austria; 2 Department of Religious Studies, University of Vienna, Vienna, Austria; 3 University Clinic for Psychiatry and Psychotherapeutic Medicine, Medical University Graz, Graz, Austria; 4 ARH: Addiction Research Hub, Grüner Kreis Ltd., Vienna, Austria; 5 Department of Human and Social Sciences, University of Salento, Lecce, Italy

**Keywords:** complex post-traumatic stress disorder, CPTSD treatment, evidence-based psychotherapy approaches, treatment outcome, treatment process

## Abstract

The treatment of patients with complex post-traumatic stress disorder (CPTSD) presents significant challenges due to the complexity and severity of the condition. Individuals who have experienced prolonged trauma exhibit, alongside post-traumatic stress disorder symptoms, disturbances in self-organization (DSO). This state of affairs not only complicates the psychotherapeutic process but also impacts the outcomes, making the treatment challenging. This article provides a narrative review of treatment approaches for CPTSD [including dialectical behavior therapy (DBT), cognitive-behavioral therapy (CBT), trauma-focused therapy (TFT), eye movement desensitization and reprocessing (EMDR), and psychodynamic therapy], with a specific focus on their outcomes and therapeutic factors. Standard evidence-based therapies (e.g., CBT, EMDR, exposure-based treatments) have been shown to be effective in reducing the core symptoms of PTSD but with more variable and often smaller effects for DSO symptoms. Phase-based approaches, including those that integrate evidence-based methods, have shown more significant results in affect regulation, self-concept, and interpersonal functioning. Psychodynamic therapies have shown enduring improvements, especially in the areas of identity and interpersonal relationships. The therapeutic alliance, trust, rupture repair, and the therapist’s empathy emerged as key components for treatment efficacy, especially in fostering safety and emotional regulation. These variables enabled corrective relational experiences and maintained engagement in therapy. These findings highlight the need for personalized, phased interventions that combine evidence-based strategies with a strong relational focus, as found in psychodynamic approaches. Addressing both symptom relief and reduction in DSO requires constant attention to the therapeutic process. The quality of the therapeutic alliance and the therapist’s responsiveness are fundamental to supporting long-term recovery. The article provides insight into the current issues associated with each treatment approach and future directions.

## 1 Introduction

Post-traumatic stress disorder (PTSD) was first introduced in the DSM-III in the 1970s to describe symptoms resulting from acute traumatic events. However, research has shown that individuals experiencing repeated and prolonged traumatic events, such as childhood abuse, neglect, domestic violence, or captivity, present with more complex symptomology that the PTSD criteria do not adequately address for diagnosis (van der Kolk et al., 2005). In response to these gaps, complex PTSD (CPTSD) was proposed by Herman (1992a), who stated that PTSD symptoms do not adequately describe the symptoms experienced by survivors of prolonged trauma. Herman (1992a) proposed an extended framework that includes disturbances in emotion regulation, consciousness, self-perception, perceived perpetrators, and systems of meaning. After this proposal, during the development of the Diagnostic and Statistical Manual of Mental Disorders (4th ed.; DSM-IV; American Psychiatric Association, 1994), the construct of Disorders of Extreme Stress Not Otherwise Specified (DESNOS) has been examined in the context of the PTSD field trials (Pelcovitz et al., 1997; Roth et al., 1997). However, CPTSD has not been adopted in the fifth edition of the DSM (DSM-5; American Psychiatric Association, 2013). In 2022, the International Classification of Diseases (ICD-11; World Health Organization, 2019) defined complex trauma as CPTSD. According to this classification, CPTSD results from exposure to an event or series of extremely threatening events that are mostly prolonged or repetitive and inescapable. Examples include repeated childhood sexual or physical abuse, torture, slavery, genocide campaigns, and prolonged domestic violence. Alongside fulfilling the primary PTSD criteria, persons with CPTSD must exhibit disruptions in self-organization (DSO), characterized by emotional dysregulation, a negative self-concept, and relational problems. These symptoms must result in considerable disruption in personal, familial, social, educational, occupational, or other critical domains of functioning (World Health Organization, 2019). Importantly, DSOs are not merely an index of greater CPTSD severity, but rather, they address qualitatively different patterns of alterations in affect regulation, self-experience, and relational functioning (Cloitre et al., 2013; Jannini et al., 2025). These patterns often reflect the developmental and interpersonal consequences of prolonged trauma and explain why CPTSD is characterized by core difficulties beyond PTSD symptoms. In line with this, empirical studies have shown that PTSD and CPTSD are characterized by different profiles, including DSO, with CPTSD associated with greater impairment. These domains are clinically relevant because they impact treatment goals and feasibility, such as safety, stabilization, and engagement, and are not systematically addressed when treatment focuses mainly on PTSD core symptoms (Ahn et al., 2025).

Complex trauma, particularly when including childhood interpersonal traumas, can profoundly affect and hinder developmental processes, attachment organization, and identity formation (Ford and Courtois, 2009; see also Farina and Schimmenti, 2025; Salvatore et al., 2024). These disruptions are linked to emotional and psychological disturbances such as affect dysregulation (Goodwin, 1988), depression, and a distorted self-image (Karatzias et al., 2017, 2018). Prolonged childhood abuse can lead to extreme dissociation and somatization symptoms. Dissociative symptoms are more prevalent in CPTSD patients than in patients with PTSD or other single traumas and are associated with CPTSD symptom severity (Dorahy et al., 2013). Furthermore, all CPTSD cluster symptoms have been found to be associated with dissociative experiences (Hyland et al., 2019). Additionally, pathological identity disturbances affect body image, internal representations of self and others, and values and ideals that provide a sense of cohesion and direction, which are gradually destroyed (Herman, 1992a).

CPTSD is frequently comorbid with several psychiatric conditions, including anxiety and mood disorders, addiction disorders (Briere and Scott, 2006; Dye, 2018; Ford and Courtois, 2009), eating disorders and self-harming behaviors (Yates et al., 2008), conduct disorders, personality disorders [especially borderline personality disorder (BPD)], dissociative disorders, and attention-deficit/hyperactivity disorder (ADHD) (Ford and Courtois, 2009; Gilbert et al., 2009). CPTSD is also associated with aggression, criminal activity, suicidal behavior, and attachment disorders (Gilbert et al., 2009). The complexity of CPTSD and these comorbidities shows the need for specialized, integrative treatment approaches.

The literature on psychotherapy for CPTSD indicates a group of evidence-based approaches, such as cognitive behavioral therapy (CBT), dialectical behavior therapy (DBT), trauma-focused therapy, and eye movement desensitization and reprocessing (EMDR). The treatment of CPTSD varies depending on the therapy’s goals. Evidence-based approaches mainly focus on reducing symptoms, while some psychotherapeutic approaches, such as psychodynamic therapy, focus on improving patients’ overall mental health and inner change, which requires more time. In literature, CPTSD treatment research focused mostly on evidence-based approaches, such as CBT, DBT, EMDR, phase-based approaches, and trauma-focused therapy. Although DBT findings are missing in CPTSD literature, existing studies focused only on PTSD symptoms but not DSO symptoms.

CBT emphasizes the identification and restructuring of maladaptive cognitions related to trauma, self-esteem, and safety. CBT may incorporate emotional control techniques and interpersonal skills development. Prolonged exposure is a structured version of CBT, which is occasionally less favored due to the potential for re-traumatization in people exhibiting DSO symptoms (Karatzias et al., 2019a,b). DBT was developed to treat BPD and initially and progressively utilized for CPTSD owing to its focus on emotion control, distress tolerance, and interpersonal efficacy. DBT assists patients in achieving stabilization prior to trauma processing and can mitigate self-harming behaviors and affect dysregulation frequently observed in CPTSD (Harned et al., 2014). EMDR targets traumatic memories with dual attention stimulation while concentrating on uncomfortable recollections. For CPTSD, EMDR is frequently administered within a phased structure: stabilization, trauma processing, and reintegration. EMDR is effective in diminishing trauma-related intrusions and is less vocally intensive (Korn, 2009). Trauma-focused therapy contains systemic protocols, such as Skill Training in Affective and Interpersonal Regulation-Narrative Therapy, which integrates emotion control, cognitive restructuring, and narrative exposure. Finally, phase-based approaches are sequential treatment models that prioritize client safety and stabilization first, followed by processing traumatic memories, and finally integration and reconnection. These approaches aim to prevent patients from becoming overwhelmed with trauma exposure before they have adequately developed resources and coping mechanisms (Cloitre et al., 2011).

Among non-evidence-based approaches, psychodynamic therapy has been proposed as a valid approach for treatment of CPTSD (Schäfer et al., 2019; Schottenbauer et al., 2008; Lampe et al., 2024). Psychodynamic therapy focuses on improving understanding of trauma’s nature and the processes of unconscious thoughts and feelings associated with traumatic events (Horowitz, 1973), brings unconscious material to awareness, provides a sense of safety, meaning, and purpose, and promotes insight into repressed thoughts and feelings that cause hyperarousal. Psychodynamic therapy also examines the therapeutic relationship for symbolic reenactment of trauma and aims to address feelings of shame and guilt (Krupnick, 2002). Besides, psychodynamic therapy focuses on interpersonal relationships, attachment patterns, and internal psychological processes (Plakun and Shapiro, 2000), and its goal is to increase unconscious awareness and reconstruct traumatic memories and meanings related to trauma, which is considered well suited for CPTSD (Herman, 1992a; Maercker et al., 2022), especially in individuals with insecure attachment or comorbid personality disorders (Høglend et al., 2006; Kernberg et al., 2008).

Despite the growing empirical interest in CPTSD, the majority of research has focused on symptom reduction rather than on exploring the therapeutic processes and relational mechanisms that facilitate recovery (Cloitre et al., 2011; Maercker et al., 2022). Furthermore, current PTSD treatment research sometimes fails to distinguish between PTSD and CPTSD (e.g., Maercker et al., 2022), often generalizing findings from PTSD populations without adequately accounting for the specific characteristics and needs of individuals with CPTSD (Maercker et al., 2022). In particular, the literature has overlooked DSO—a key feature of CPTSD—with few empirical reviews addressing DSO-related treatment outcomes, as well as the impact of relational and therapist-related variables. While evidence-based treatments such as CBT, DBT, and EMDR are well studied, other approaches such as psychodynamic therapy are underrepresented despite their historical relevance and clinical applicability in addressing CPTSD (e.g., Schottenbauer et al., 2008; Lampe et al., 2024). Given the relational nature of CPTSD and the centrality of DSO, more attention should be given to approaches such as psychodynamic therapy, which explicitly address identity, affect regulation, and interpersonal dynamics. More specifically, psychodynamic therapy may be effective in addressing DSO due to its focus on attachment patterns, mentalizing, and reshaping internalized relational patterns, all of which are closely linked to emotional dysregulation and interpersonal difficulties (Fonagy et al., 2002; Schore, 2003; Levy et al., 2015; Kernberg et al., 2008; Cloitre et al., 2014). A broader, more integrative understanding of the therapeutic landscape for CPTSD is needed, considering not only outcomes but also therapeutic conditions and relational dynamics that facilitate change. To this end, the present study provides a narrative (non-systematic) review of current psychotherapeutic approaches to the treatment of CPTSD, with a specific focus on treatment outcomes and therapeutic factors. While a systematic review is beyond the scope of this article, a targeted, structured literature search was conducted in major databases (EBSCO, PsychINFO, PubMed, ScienceDirect, PsychARTICLES, ProQuest, and Academic Search Premier). Search terms included “treatment approaches for CPTSD,” “psychotherapy of CPTSD,” “psychodynamic therapy for CPTSD,” “humanistic therapy for CPTSD,” “efficacy research for CPTSD,” “outcome research for CPTSD,” “process research for CPTSD,” and “therapeutic factors for CPTSD.” The focus was limited to peer-reviewed journal articles written in English that addressed the outcome of psychotherapy for CPTSD and the therapeutic factors involved in the therapeutic process. Studies involving children, group or expressive therapies, or focusing exclusively on specific techniques (e.g., single-session tasks) without reference to a structured psychotherapeutic model were excluded. In line with the aims of this narrative review, we did not implement any protocol for study selection and data extraction and synthesis, nor any formal risk-of-bias assessment.

## 2 Therapeutic outcomes

### 2.1 Phase-based approaches

Regarding the multidimensional symptom structure of CPTSD, phase-based approaches have been extensively considered as treatment frameworks that prioritize safety, stabilization, affect regulation, and interpersonal functioning before trauma processing (Herman, 1992a,b; Cloitre et al., 2011; Courtois and Ford, 2013).

The International Society for Traumatic Stress Studies (ISTSS) promoted an expert clinician survey on best practice for CPTSD treatments (Cloitre et al., 2011). A team of experts evaluated the most appropriate treatment approach and specific interventions for CPTSD, including efficacy, safety, and acceptability. The distribution of experts’ ratings of overall approaches was divided into three categories comprised by first-, second-, and third-line approaches and interventions. The experts endorsed a sequential and multicomponent approach oriented toward the patient’s most prominent symptom domains as a first-line approach, followed by second-line approaches focusing primarily on coping skills or a combination of skills and processing, and finally third-line approaches focusing primarily on memory processing (Cloitre et al., 2011). Regarding the initial phase of the treatment, psychoeducation and emotional regulation strategies were identified as first-line interventions across symptom domains, while trauma memory narration and cognitive restructuring were frequently endorsed as core interventions, depending on the symptom target domain. Regarding acceptability, first-line interventions were psychoeducation, emotion regulation strategies, and anxiety/stress management. Most other interventions (i.e., interpersonal/social skills, meditation/mindfulness, narration of trauma memory, and case management) fell between first- and second-line interventions. Finally, bilateral stimulation and sensorimotor/movement strategies were considered second-line interventions. Moreover, experts rated individual therapy as a first-line treatment modality for both the initial phase and trauma memory processing. Individual plus group settings were rated between first- and second-line treatment modalities, while open group formats and self- were rated between second- and third-line modalities. Nevertheless, it has been emphasized that safety and affect regulation should be prioritized before exposure-based work. This ensures adequate stabilization and pacing before trauma processing, which prevents distress, traumatization, or a failure to enhance overall quality of life (Cloitre et al., 2010; Courtois, 2004; van der Kolk et al., 2005; Palic and Elklit, 2011).

A phase-based approach may be integrated into other approaches. Cloitre et al. (2002) conducted a randomized clinical trial (RCT) and developed a cognitive-behavioral strategy that was phased in and sequential, whereby exposure treatment was preceded by Skills Training in Affective and Interpersonal Regulation (STAIR). They mentioned patients who completed the Skill Training in Affective and Interpersonal Regulation (STAIR) exposure treatment showed significant improvements in affect regulation, interpersonal functioning, and PTSD symptoms, and these improvements were maintained during follow-up. The authors concluded that adding an emotional and interpersonal stabilization phase to standard trauma-focused cognitive-behavioral therapy is beneficial in complex groups. After an extended period of research, Cloitre et al. (2010) proceeded to expand their findings by evaluating STAIR exposure with PE treatment in a group of women who had been subjected to child abuse and subsequently developed chronic PTSD. Participants who were treated with STAIR therapy had a higher chance of having higher remission and no longer meeting PTSD criteria at the end of treatment than those treated with PE alone. A new modular person-centered therapy called Enhanced Skills Training in Affective and Interpersonal Regulation (ESTAIR) has been proposed that adopts the principles of STAIR but also works on the impact of trauma on relationships and emotional distress in the present, which has an effect on quality of life (Karatzias et al., 2023). In line with this, a pilot RCT comparing ESTAIR with treatment as usual in CPTSD showed significant reduction in both PTSD symptoms and DSO [assessed with the International Trauma Questionnaire (ITQ)] (Karatzias et al., 2024). Moreover, an umbrella review found that phase-based approaches showed greater efficacy than single-phase approaches. The review showed that multicomponent interventions, including phase-based approaches, are beneficial for treating PTSD core symptoms in CPTSD, impacting anxiety, sleep, and depressive symptoms (Billings and Nicholls, 2025). These findings support the idea that treating patients with CPTSD symptom patterns by focusing on their emotion regulation and interpersonal function issues before introducing exposure-based therapies may result in significant improvements in treatment outcomes (Cloitre et al., 2011).

In summary, the above findings converge on the idea that a phase focused on emotional regulation and relational competencies, added to the beginning of treatment, seems to be associated with better outcomes. These outcomes include sustained changes at follow-up and higher rates of remission compared to exposure therapy alone. These findings are consistent with the STAIR model for treating early interpersonal trauma (Cloitre et al., 2002; Cloitre et al., 2010) and with the results of the ISTSS clinical survey on best practices for CPTSD (Cloitre et al., 2011). Interestingly, in support of the clinical relevance of addressing DSO, a recent RCT on adult survivors of childhood abuse showed that EMDR or narrative therapy followed by STAIR decreases PTSD symptoms and improves DSO’ affective dysregulation and interpersonal problems (Wigard et al., 2024). Therefore, when the clinical target includes DSO, it may be advisable to first focus on stabilizing and enhancing emotional regulation and interpersonal functioning, and only then carry out trauma-focused interventions.

### 2.2 Evidence-based approaches

While phase-based models prioritize preparatory stabilization, evidence-based interventions (e.g., CBT, EMDR, DBT, trauma-focused therapy) were the most explored treatments in literature for PTSD (Ursano et al., 2004) and have increasingly been extended to CPTSD populations (Bisson and Andrew, 2007; Cloitre et al., 2011; Karatzias et al., 2019a,b).

People with more complicated pathologies or multiple traumas may not necessarily benefit from typical cognitive behavioral therapy (CBT) that aims to treat symptoms regarding a specific traumatic incident (Lonergan, 2014), because CPTSD includes affective instability, dysphoria, and interpersonal dysfunction that need different treatment compared to PTSD (van der Kolk et al., 2005). Based on this, efficacy research examined exposure-based CBT with emotional and relational skills training. In the treatment literature, there has been discussion over whether exposure-based cognitive behavioral therapy is suitable for people with complex symptoms (Palic and Elklit, 2011; van der Kolk et al., 2005). Conversely, some experts argue that if dissociative symptoms, interpersonal problems, and emotional instability are neglected before exposure treatment, beneficial outcomes may be limited (Bisson and Andrew, 2007). In line with these promising results, two meta-analyses (Mahoney et al., 2019; Karatzias et al., 2019a,b) indicate that trauma-focused treatments, with or without phased interventions, might be beneficial for individuals with CPTSD symptoms. More recently, a systematic review showed that different psychological interventions can reduce symptoms related to CPTSD and improve areas that overlap with DSO (e.g., affect dysregulation and interpersonal problems), while emphasizing the heterogeneity of the findings and the variability in how these outcomes are assessed and reported (Ahn et al., 2025).” However, comparative RCT directly contrasting phase-based and trauma-focused treatments for CPTDS remain limited, although emerging evidence is starting to address this gap. For example, one of the first studies to explore trauma-focused treatment outcomes for CPTSD found that trauma-focused therapy decreased PTSD and CPTSD symptoms in an inpatient setting (Voorendonk et al., 2020). Earlier literature has also suggested that trauma-focused treatments may decrease PTSD symptoms but not necessarily alleviate other mental health difficulties (Nickerson et al., 2011). These studies present promising results regarding the efficacy of trauma-focused treatment approaches on DSO symptoms, including affect regulation, negative self-concept, relational problems, and comorbidity in CPTSD. However, these findings require further evidence.

A smaller, yet clinically relevant, strand is represented by humanistic-experiential approaches, especially emotion-focused therapy for trauma (EFTT). Some RCTs have shown that adults with a history of child abuse and neglect may benefit from EFTT in terms of reduced symptoms and increased of self- and interpersonal functioning. However, these findings did not strictly refer to ICD-11 CPTSD diagnosis and outcome measures (Paivio and Nieuwenhuis, 2001; Paivio et al., 2010).

A limited number of studies have explored the use of CBT treatment, excluding phase-based approaches, in the context of CPTSD among individuals exposed to chronic war trauma, such as refugees and political prisoners (Cloitre et al., 2009). These studies have indicated that CBT may hold potential benefits for this specific population. However, there remains a significant gap in research addressing individuals with a history of multiple traumas, including sexual, physical, and emotional abuse; neglect; domestic violence; and partner violence. These populations have been under-researched and have been utilizing inappropriate diagnostic and measurement tools for CPTSD (Ross et al., 2021). Niemeyer et al. (2022) conceptualized these populations of individuals as at-risk groups for complex traumatization, and the available evidence suggests that trauma-focused CBT and EMDR can be effective in these groups. Further efficacy studies should focus on individuals properly diagnosed with CPTSD who have experienced relational traumas, as mentioned above.

A systematic review and meta-analysis was conducted to examine the comparative efficacy of cognitive behavioral therapy (CBT), eye movement desensitization and reprocessing (EMDR), and exposure therapy for complex posttraumatic stress disorder (CPTSD). The findings indicated that EMDR exhibited a modest advantage over CBT in terms of reducing PTSD symptoms. However, no significant differences were observed between CBT, exposure alone, and EMDR in terms of DSO symptoms. The findings of the present study indicated that the effectiveness of evidence-based therapies, such as cognitive behavioral therapy (CBT), exposure alone, and eye movement desensitization and reprocessing (EMDR), was lower than that of other psychological treatments (Karatzias et al., 2019a,b). Authors stated that the developmental stage of psychological trauma and childhood traumas may influence the course of evidence-based treatments, which is related to less significant impact on CPTSD symptoms. Furthermore, it was indicated that none of the therapies—exposure, cognitive reappraisal, or EMDR—is enough to treat one symptom cluster for CPTSD. Patients with CPTSD who have childhood traumas had less improvement in those treatments (Karatzias et al., 2019a,b). In parallel to this result, a quantitative review article compared the effectiveness of CBT treatment between PTSD patients who have childhood abuse and CPTSD patients who have childhood abuse (Dorrepaal et al., 2014). It was found that CBT is less helpful for CPTSD patients compared to PTSD patients when they have childhood abuse.

Overall, these findings suggest that evidence-based approaches may be effective. However, the extent and clinical domains of improvement do not always overlap between PTSD and CPTSD, especially when the clinical profile includes developmental or interpersonal trauma and pronounced DSO symptoms (Lonergan, 2014; Ross et al., 2021). In line with this, the quantitative reviews described above suggest that evidence-based approaches can be useful, though they have significant limitations related to sample heterogeneity, measures, and targeted outcomes. In some studies, the risk is that the overall outcome is more related to core PTSD symptoms than to DSO domains. For example, the lower efficacy of CBT in cases of childhood abuse is a clinically relevant indicator that it may be advisable to integrate specific modules for emotional and interpersonal regulation in more complex subsamples (Dorrepaal et al., 2014) or adopt a treatment sequence that does not reduce DSO symptoms as a secondary outcome.

### 2.3 Psychodynamic approaches

Beyond symptom-focused and skill-based approaches, CPTSD conceptualization stems from chronic or repetitive relational traumas, disruptions in attachment, and disturbances in self-structure and affect regulation. Psychodynamic therapy focuses on unconscious processes, interpersonal relationships, mentalization, and meaning-making as core mechanisms for change (Schottenbauer et al., 2008; Bateman et al., 2018).

A naturalistic study was conducted to compare psychodynamic therapy to prolonged exposure for CPTSD patients. PTSD symptoms were significantly reduced, and emotion regulation skills increased in patients whose therapists used a psychodynamic approach, and attention biases and implicit memory slightly improved. However, prolonged exposure did not significantly reduce symptoms; instead, the patient showed increased attentional biases and worsening emotion regulation symptoms.

Multimodal psychodynamic therapy for CPTSD was explored in an inpatient rehabilitation center. They measured PTSD and DSO symptoms independently, and the results indicated a significant decrease in trauma-related symptoms and DSO symptoms; however, compared to PTSD symptoms, patients reported more improvement in DSO symptoms (Lampe et al., 2024). In the study, they shared that this result is parallel to previous findings (Schäfer et al., 2019) for the treatment of CPTSD. They showed multimodal psychodynamic treatment also reduced symptoms of depression, anxiety, and somatization. In that study, patients’ negative self-concept and interpersonal relationships showed the most reduction of symptoms, while intrusive symptoms and ongoing feelings of increased present danger showed the lowest reduction of symptoms. On the other hand, affect dysregulation was still high at the end of therapy. Further, Schottenbauer et al. (2008) indicated that psychodynamic therapies are effective for treating CPTSD patients because they enhance their self-esteem, self-reflection, ability for mentalization, and internalization of secure attachment. It was noted that treating mentalizing issues in addition to epistemic mistrust is essential to transformation and recovery (Bateman et al., 2018, 2023).

Furthermore, longitudinal experimental studies have demonstrated the sustained benefits of psychodynamic treatment for CPTSD. It was demonstrated that these improvements persist for years following the conclusion of therapy, suggesting that symptoms continue to decrease even after the cessation of treatment (Blomberg et al., 2001; Sandell et al., 2000). These findings demonstrate that psychodynamic/psychoanalytic therapy induces changes within the person or self, which is related to the continuation of improvement even after years of therapy have ended. Another study of psychodynamic therapy found CPTSD patients’ functioning had significantly improved by the end of therapy and at the 12-month follow-up point and that their PTSD and depressive symptoms had decreased by the end of therapy and follow-up assessment. Furthermore, an increase in patients’ hope has been observed (Levi et al., 2017). A parallel set of findings emerged from a separate observational study. In this study, multimodal psychodynamic inpatient rehabilitation treatment was administered to patients with CPTSD for a period of 6 weeks. A follow-up assessment was conducted after a period of 28 months had elapsed, corresponding to the completion of 6 weeks of treatment (Riedl et al., 2025). It was found at the end of treatment that 60% of patients were no longer diagnosed with CPTSD, and in the 28-month follow-up, this remained stable with improved epistemic trust and reduced epistemic credulity. In follow-up, they observed DSO symptom reduction remained stable, whereas PTSD symptoms decreased. Moreover, it was found that patients reported high satisfaction with the treatment. Brom et al. (1989) supported that in their controlled outcome study by showing brief psychodynamic therapy with traumatized patients had a positive impact and improvement after treatment had ended compared to hypnotherapy and trauma desensitization. Krupnick (2002) added that brief psychodynamic therapy worked for PTSD, and in a follow-up session one and a half years after treatment ended, the patient’s situation was okay, even better; however, this treatment might have implications for the complexity of CPTSD because, as mentioned in the study, this treatment might be beneficial for high-functioning patients that want a treatment that includes self-analysis, introspection, and discovering meanings.

Despite the lack of research exclusively focused on CPTSD, the psychodynamic approach often addresses essential elements, including self-concept and relational disruptions, which are included in DSO symptoms. In psychodynamic therapy literature, studies mostly examined PTSD. However, those studies include prolonged exposure to trauma, self-concept, and interpersonal problems. Moreover, the diagnosis of CPTSD is either not mentioned or mentioned but not included properly. Another issue is that literature does not make a distinction between single trauma and complex trauma (CPTSD). Due to the implications of complex trauma, some clinicians and researchers believe that psychodynamic therapy may be a clinically relevant treatment option for clients with CPTSD exhibiting prominent self- and interpersonal difficulties (D'Andrea and Pole, 2012).

These findings suggest that psychodynamic therapy can be effective in targeting not only PTSD symptoms but also DSO domains, especially with regard to self-concept and relational problems. However, other aspects, such as affect dysregulation and some specific PTSD symptoms, may persist at the end of treatment (e.g., Lampe et al., 2024). Moreover, longitudinal data show favorable long-term outcomes in terms of stable DSO improvement and, in some cases, further reduction of PTSD symptoms, increased epistemic trust, and greater treatment satisfaction (e.g., Riedl et al., 2025). However, this evidence is limited and inconsistent (e.g., Brom et al., 1989; Krupnick, 2002), with only a few studies systematically evaluating the efficacy of psychodynamic therapy on DSO. For this reason, even though the rationale of psychodynamic therapy appears to align with the developmental and interpersonal nature of CPTSD, more robust comparative studies are needed.

### 2.4 Integrative approaches

Given the limitations of a single psychotherapy approach, integrative approaches have emerged due to the complexity of CPTSD as more individualized treatment (Schottenbauer et al., 2006; Cloitre et al., 2011; Karatzias et al., 2023).

The integration of CBT with psychodynamic or interpersonal therapy was explored in an experimental study. CBT-oriented therapists have demonstrated their integration of interpersonal and psychodynamic approaches within CBT for patients suffering from trauma and PTSD (Schottenbauer et al., 2006). Although the study did not include DSO symptoms for CPTSD, it was mentioned that for prolonged trauma or complex trauma, interventions focused on lowering PTSD symptoms alone might not be enough. The limited impact of CBT alone also supports the importance of an integrative and individualized treatment approach for CPTSD.

Independent from the therapeutic approach, due to the nature of CPTSD, treating CPTSD itself is very complex. Considering CPTSD treatment with a comorbid disorder, it might require a distinct and more targeted approach. The treatment of CPTSD with comorbid disorders was addressed only in a few outcome studies. A case study examined CPTSD patients comorbid with BPD and dependent personality disorder who were treated with metacognitive interpersonal therapy; they observed hyperarousal, dissociation, and dysregulation were decreased (Popolo et al., 2025). Another case study was done about EMDR treatment for CPTSD comorbid with BPD (De Jongh and Hafkemeijer, 2024), involving 10 EMDR sessions held over the course of 5 weeks. Findings showed that, at the end of treatment, the patient did not meet the criteria for CPTSD and BPD.

Despite growing empirical interest, research on CPTSD treatment is limited. Many studies focusing on integrative approaches have lacked distinguishing PTSD and CPTSD, consistent diagnosis, or adequate diagnostic tools. RCT studies comparing psychodynamic, CBT, and integrative treatment approaches are insufficient. Further, dissociation is not addressed adequately in many outcome studies, which is found to be a crucial factor affecting the treatment process and outcome to focus on dissociative symptoms in the treatment of extensive trauma histories (Cook et al., 2004). Yet, research suggested that a psychodynamic and integrative therapeutic approach might provide broader and lasting effects for CPTSD patients compared to trauma-focused therapies alone (Horesh and Lahav, 2024). Overall, these findings suggest that integrating different approaches should account for the clinical heterogeneity of CPTSD (e.g., comorbidity, dissociation, relational instability, and setting vulnerability) and the need for more individualized and articulated treatments (Schottenbauer et al., 2006; Cloitre et al., 2011; Karatzias et al., 2023). Specifically, in cases of prolonged and/or complex trauma, reducing PTSD symptoms alone may not address the specific needs of CPTSD patients (Schottenbauer et al., 2006). Furthermore, existing evidence lacks the methodological rigor of randomized controlled trials (RCTs) and remains limited, often relying on case studies, particularly regarding comorbidities. However, it suggests the potential benefits of targeted and personalized approaches (Popolo et al., 2025; De Jongh and Hafkemeijer, 2024).

## 3 Therapeutic factors

Factors contributing to the outcome of therapy can be listed as therapist factors, patient factors, therapeutic relationship, therapeutic attunement, expectations, motivation, insight, self-efficacy, and emotional experience (Ackerman and Hilsenroth, 2001; Bailey and Ogles, 2023; Laska et al., 2014). Consistent with the broader psychotherapy process literature, the factors discussed in this section can be understood as largely transtheoretical (i.e., common processes) that are described and operationalized in different ways across the different approaches considered so far (for a general discussion, see Wampold and Imel, 2015). Accordingly, the concepts we refer to are drawn from different therapeutic schools with the aim of addressing change processes that are school-independent rather than implying that any single school uniquely accounts for them. At the very general level, research on the therapeutic factors of CPTSD treatment is limited.

### 3.1 Patient-related factors

Patient-related factors such as attachment organization, emotion regulation capacity, readiness, insight and motivation, coping mechanism, trauma history, and comorbidities have been found to significantly affect outcome. Studies of different psychotherapy approaches found an association between attachment style and both alliance and outcome (e.g., Diamond et al., 2003, Fonagy et al., 1996). Understanding the attachment organization of a patient helps to build and maintain a therapeutic relationship with a CPTSD patient and understand the patient’s problems, their coping mechanisms, and their emotion regulation as well (Pearlman and Courtois, 2005). It was shown that a patient’s attachment organization is related to the quality of the therapeutic relationship (Diamond et al., 2003) and therapy outcome (Fonagy et al., 1996). Dismissing attachment organization was found linked to CPTSD, whereas fearful attachment organization was found correlated to higher DSO symptoms, and secure attachment organization was found associated with fewer disturbances in DSO symptoms (Karatzias et al., 2021). Besides, anxious and avoidant attachment organizations were found to be differentiating factors for CPTSD (Karatzias et al., 2018). Psychotherapy research has shown that attachment organization changes through therapy (Schottenbauer et al., 2008). Furthermore, psychotherapy approaches focusing on early development and therapeutic alliance may be more relevant for those who have insecure attachment and early trauma histories (Høglend et al., 2006).

Patient’s readiness, insight, motivation, interpersonal skills, and emotion regulation abilities during the process affect the therapeutic relationship and therapy outcome (Cloitre et al., 2010; Cloitre et al., 2004; Dorrepaal et al., 2014; Karatzias et al., 2019a,b). Trauma history and comorbidities were found linked to treatment outcome. A meta-analysis was done to understand predictors of outcome for trauma-focused therapy for PTSD (Keyan et al., 2024). It was shown that trauma history, trauma type (single or multiple, prolonged), comorbidity (depression, alcohol addiction, personality disorders), sleep problems, and treatment modality affect treatment outcome. Given that, it was mentioned that trauma history, symptom severity, comorbidity, and prolonged and multiple traumas caused poor treatment outcomes. Therefore, this shows the importance of evaluation of the patient and diagnosis before beginning therapy. This is in line with research that found that broader evaluation, comprehensive assessment (Cloitre, 2021), and early and accurate diagnosis (Hyland et al., 2020; Karatzias et al., 2021) of CPTSD patients lead to better outcomes, lower dropout rates, more effective therapy goals, and long-term treatment efficacy. Therefore, evaluation of the patient and diagnoses are very important for the treatment process and outcome, which is in line with literature (Krupnick, 2002), which indicated that taking trauma history, stressful life events, and losses is important in evaluation; it might also show where there are unresolved feelings that might be reactivated while working on trauma in psychodynamic therapy. The evaluation must include assessment of the patient’s strengths and weaknesses that focuses on coping mechanisms and emotional states that show how the patient deals with the outside world.

### 3.2 Therapist-related factors

Therapist-related factors such as empathy, holding and containing, responsiveness, therapeutic presence, and the capacity to repair ruptures have been found to be significantly associated with outcome. Research highlights the importance of a therapist’s personal and relational qualities for effective treatment, although research findings are limited for CPTSD treatment. Empathic attunement, responsiveness, and the ability to regulate in-session affect are considered pivotal qualities of therapists, though they can be described and termed in different ways across different therapeutic orientations. Psychodynamic approaches define these qualities as holding and containing, as well as managing transference and countertransference processes (Courtois, 2004; Davies and Frawley, 1994; Pearlman and Saakvitne, 1995; Schwartz, 2000). CBT approaches conceptualize these qualities in terms of collaborative empiricism and guided discovery (Cloitre et al., 2004; Laska et al., 2014). Dialectical behavior therapy (DBT) describes these qualities as validation and maintaining a dialectical stance (Harned et al., 2014). EMDR defines these qualities as maintaining dual attention within a safe therapeutic environment (Korn, 2009; De Jongh and Hafkemeijer, 2024). In trauma therapy, empathy is a crucial component for therapeutic relationships and effective treatment. However, empathy in trauma therapy involves not only witnessing patients’ trauma and helping acknowledge trauma but also involving transference and countertransference (Pearlman and Saakvitne, 1995; Toporek et al., 2025). Further, through the therapist’s holding and containing, the patient develops trust, feels safe, and reshapes their internal relational model. This process helps patients experience intimacy without fear or denying the need for care and love, which helps them have a corrective experience; as a result, they can build and maintain healthier and more independent relationships (Papa et al., 2024).

Besides, a therapist’s capacity to identify and repair ruptures is crucial in trauma therapy, especially in CPTSD treatment. Levi’s (2019) case study on psychodynamic therapy for CPTSD showed that the therapist’s position as containing, holding, and being present helped the patient to feel hope and see the potential for change and growth. Levi (2019) stated that containing and holding a therapist enables the patient to feel emotional and deep and experience another way of being. Research about the relational treatment approach to trauma noted that when working with CPTSD, therapists must be aware of the countertransference and manage it well by using it to understand patients’ shifting states and handle their emotions, which may stem from patients’ real issues or projective identification or provocation (Davies and Frawley, 1994; Pearlman and Saakvitne, 1995; Schwartz, 2000). Levi (2019) mentioned that therapists who notice countertransference processes and lower the risk of drop-out by building strong therapeutic alliances, exploring pain and fear while maintaining positive and hopeful attitudes, and offering encouraging therapeutic processes find balance between possibilities (hopes) and limitations (realistic hopes). The relationship with the therapist and the therapist’s responsiveness were found crucial in recovery (Matheson and Weightman, 2021). Further, the therapist’s personality and presence were found to be related to better outcomes. The therapist must provide unconditional acceptance and allow the patient to use the therapist’s ability to bring new hope and growth to the therapy process. In addition to that, therapists must deal with patients’ fear and despair as well as analytical processes without disappearing (Mitchell, 1993; O’Hara, 2011).

### 3.3 Therapeutic relationship and therapeutic alliance

Therapeutic relationship, alliance, and trust are regularly regarded as essential for a positive therapy outcome. Therapeutic relationships and alliances include building therapeutic relationships and trust and maintaining them by repairing therapeutic ruptures, causing corrective emotional experiences, working on relational reenactments and transference, and containing during crisis moments. Forming therapeutic relationships with traumatized patients is addressed by many clinicians and researchers (Courtois, 1988, 1999; Dalenberg, 2000; Davies and Frawley, 1994; Herman, 1992a,b; Pearlman and Saakvitne, 1995; Schwartz, 2000). Findings showed that mechanisms associated with trauma might affect therapeutic alliance.

Establishing trust and a therapeutic relationship with severely traumatized patients might take a long time and be very difficult (Papa et al., 2024; Pearlman and Courtois, 2005). Severely traumatized individuals cannot show progress if trust in the therapeutic relationship has not been adequately established and emotion regulation has not been sufficiently lowered, given the comparatively brief six-week therapy period (Lampe et al., 2024). Another study was done with young women who have CPTSD. They shared that it took many sessions in the beginning to trust the therapist (Beaton and Thielking, 2019). Building a strong therapeutic relationship enhances emotion regulation and coping abilities, which are all key components of different psychotherapy orientations (Wöller et al., 2012, 2020). A study explored CPTSD patients’ views about therapy; findings showed that establishing trust in therapeutic relationships was crucial to recovering from trauma because it enables people to talk about traumas and experience a sense of relief and release. Besides, it was found to be the basis for rebuilding other relationships. The therapeutic relationship is an important factor in trauma treatment, specifically in complex trauma. Research was done with therapists and showed that complex trauma patients heal in relational contexts because they were traumatized in relationships (Toporek et al., 2025). A case study based on qualitative analysis explored relational dynamics in therapeutic relationships with CPTSD patients (Papa et al., 2024). It was reported fear of intimacy and complex trauma might be a risk factor for therapeutic alliance. They mentioned when the therapist took on a caring role and the therapeutic relationship progressed, the patient experienced a fear of intimacy that was triggered by a threat of losing the therapist or being abandoned because of an interruption in therapy due to the therapist’s pregnancy.

Repairing therapeutic ruptures is very important, especially with patients who have a history of repeated relational trauma/complex trauma because they do not believe relationships can be repaired. A case study based on qualitative analysis found repairing ruptures is an ongoing process (Papa et al., 2024). A case study was done with a CPTSD patient comorbid with BPD that showed that when there is an alliance rupture, repair is required to reestablish self-regulation and emotion regulation (Popolo et al., 2025). They noted that therapists must be aware of the activation of maladaptive relational patterns when ruptures are repaired and therapeutic relationships restored; there will be a place for self-regulation and understanding the triggers for maladaptive relational patterns. It was mentioned in a case study that the dynamic between patient and therapist, with all disconnections, connections, and repairs, mirrors the patient’s attachment (Papa et al., 2024).

A good therapeutic relationship, trust, safety, and repairing therapeutic ruptures help patients to have corrective emotional experiences. Case studies indicate that therapeutic relationships become corrective experiences; patients’ representations of the self and others are replaced by healthier representations, such as going from being needy and undeserving of care and love to deserving of care and love and having a sense of inner strength and worth. Therefore, working on relational reenactments, transference, countertransference, past traumatic experiences, therapeutic relationships, repairing ruptures, and using self-regulation techniques helps patients to gain awareness and self-control (Popolo et al., 2025). Containment during a time of crisis was found in a case study that enables a patient to experience another way of being when there are no words or interpretations (Levi, 2019).

In CPTSD treatment, these relational dynamics are highlighted not only in psychodynamic therapy but also in phase-based, evidence-based, or integrative approaches that prioritize safety, emotional regulation, and interpersonal functioning (Horesh and Lahav, 2024). For example, alliance building and rupture-repair processes are explicitly mentioned in evidence-based and phase-based CPTSD treatment approaches. These approaches emphasize collaborative case formulation, ongoing attention to engagement and disengagement processes, and the use of grounding or stabilization strategies when clients’ dysregulation threatens treatment progress (Cloitre et al., 2004, 2011; Papa et al., 2024).

### 3.4 Processes supporting agency and self-organization

Fostering agency and control helps to reduce reenactments of victimization. During the treatment it is important to give the patient a sense of control and enhance their sense of agency, because childhood trauma survivors were put in a passive position by their aggressors, which caused them to undermine their sense of entitlement and agency (Van Nieuwenhove and Meganck, 2020). This is also important as therapists to prevent the retraumatization through relational insight (Van Nieuwenhove and Meganck, 2020). Therapists should have insight about patients’ interpersonal patterns to prevent repetition of these patterns and maintain the therapeutic relationship. Supporting mentalization and reflective functioning helps patients to develop new relational experiences, which also gives them the opportunity to revise and rework deeply those interpersonal patterns that allow patients to see themselves, others, and the world in a different way (Van Nieuwenhove and Meganck, 2020).

### 3.5 Structural/relational variables

Therapy duration and intensity were explored in the literature. For the length of treatment of CPTSD, it has been mentioned in several studies that CPTSD requires longer treatment because of the large symptom scale, including self-identity, emotion regulation, and relational deficits (Beaton and Thielking, 2019; Cloitre et al., 2004; Matheson and Weightman, 2021). It was reported that participants who were in weekly therapy shared that it was necessary for them to be in therapy much longer than a year (Beaton and Thielking, 2019). Patients’ views were also about the length of therapy; at least a year of psychotherapy was found necessary for the treatment of CPTSD (Matheson and Weightman, 2021). Setting and maintaining boundaries were found challenging during the CPTSD treatment process (Pearlman and Courtois, 2005). Another crucial component in trauma therapy is dissociation and the patient’s ability to stay in the here and now. Notably, the management of dissociation and anchoring to the present moment are considered relevant across different therapeutic orientations. These issues are typically addressed through stabilization prior to trauma processing (in phase-based approaches), grounding and resource-oriented strategies, and careful pacing of exposure (in trauma-focused CBT), as well as dual attention (in EMDR) when arousal becomes excessive or overwhelming (e.g., Cloitre et al., 2011; Ford et al., 2005; Korn, 2009). Moreover, in a psychodynamic case study, Henley (2023) explored dissociative reactions during the therapy process, findings showed that psychodynamic techniques aimed at highlighting unconscious forms of traumatic stress and bringing them to consciousness generate emotional engagement. However, except for this study, dissociation is not addressed or explored in process or process and outcome studies to focus on dissociative symptoms in the treatment of extensive trauma histories (Cook et al., 2004).

Termination dynamics is an important concept that should be considered carefully in the treatment of CPTSD. Beaton and Thielking (2019) mentioned that CPTSD patients’ internal working models are activated in therapy, and during the termination, they might want to end the therapy process early or might explode at the time of separation. Because termination affects special issues and evokes feelings of abandonment, grief, fear, and loss of security (Herman, 1992b), it is recommended to leave an option for return for continuation of therapy or even for check-in during the termination of therapy.

## 4 Discussion

This narrative review addressed treatment outcomes related to different therapeutic approaches for CPTSD and important therapeutic factors during the treatment process of CPTSD. The article highlights three main findings related to the treatment complexity of CPTSD that is yet insufficiently explored in clinical research and practice: (1) Evidence-based psychotherapy approaches reduce PTSD symptoms but are less effective for DSO symptoms; (2) emerging evidence supports phase-based and psychodynamic approaches; and (3) therapeutic process factors such as therapeutic relationship and alliance, trust, and repairing ruptures play a crucial role but are, however, understudied in literature.

### 4.1 Treatment outcomes: toward a personalized and integrated approach

Empirical evidence suggests that evidence-based treatments such as CBT, DBT, trauma-focused therapy or trauma-focused CBT, and EMDR are effective at reducing mostly PTSD symptoms (Ahn et al., 2025; Karatzias et al., 2019a,b; Mahoney et al., 2019); however, they are limited in treating DSO symptoms (Dorrepaal et al., 2014; Karatzias et al., 2019a,b), which are less frequently addressed in clinical trials or treatment protocols (Ahn et al., 2025; Maercker et al., 2022). In this context, some available evidence – though still limited – on humanistic-experiential approaches, such as EFTT, suggests a possible contribution on targets consistent with DSO, but more studies are needed that explicitly assess CPTSD according to ICD-11 (Paivio and Nieuwenhuis, 2001; Paivio et al., 2010). Consequently, the efficacy of current PTSD treatment approaches is sometimes limited in cases of CPTSD, though recent studies have shown that integrating skills-based modules (see phase-based approaches) within trauma-focused approaches can enhance improvements also in DSO domains (Karatzias et al., 2024; Wigard et al., 2024).

Phase-based treatments, whether administered individually or in combination with exposure therapy, have shown improvements in emotion regulation and interpersonal functioning. However, their limitations in addressing relational traumas underscore the value and depth of evidence-based treatment modalities. De Jongh and Hafkemeijer (2024) noted that the phase-based approach should be converted to a more personalized approach to treat CPTSD. This is also in line with other research that explored commonalities and differences between therapeutic approaches with five case studies (Farina et al., 2025). It emerged that CPTSD treatment requires individualized treatment tools and strategies, modifying treatment to different patient presentations rather than adopting a treatment protocol for all patients. It was also noted that patients come to therapy without a request to treat trauma or an awareness of how traumas cause their current suffering. Horesh and Lahav (2024) stated that traumas cannot be treated in the beginning of therapy or until patients realize the connection between symptoms and traumatic memories. Importantly, evidence-based treatments for CPTSD are typically delivered using structured protocols that promote treatment consistency and dissemination (Cook et al., 2004; Schottenbauer et al., 2006). In contrast, psychodynamic approaches tend to emphasize shared meaning-making, developmental history, and recurring relational patterns (Davies and Frawley, 1994; Spermon et al., 2010; Wöller et al., 2012). However, these emphases are not mutually exclusive and are increasingly combined in phase-based, integrative, or modular approaches for treating CPTSD (Cloitre et al., 2002, 2011; Karatzias et al., 2023; Farina et al., 2025; De Jongh and Hafkemeijer, 2024). The empirical evidence for the treatment of CPTSD with psychodynamic therapy remains sparse and is characterized by lower methodological quality (e.g., lack of RCTs) compared to the evidence for other approaches reviewed in this paper (e.g., trauma-focused CBT) (Bradley et al., 2005; Lampe et al., 2024; Riedl et al., 2025). Nevertheless, some available findings suggest that psychodynamic therapy can produce clinically meaningful outcomes, especially in longer treatments targeting broader personality and relational functioning (Lampe et al., 2024; Riedl et al., 2025). Beyond the discussion of which approach is more suitable for treating CPTSD, the evidence reviewed in this paper suggests that tailoring interventions for CPTSD leads to better treatment outcomes due to the disorder’s complexity.

Overall, these findings suggest that CPTSD requires personalized, flexible treatment rather than rigid protocols. There is a growing consensus that CPTSD patients require individualized therapy since their developmental histories and suffering are different. The therapeutic relationship should be the foundation of treatment, with the objective of ensuring treatment adherence, preventing patient dropouts, and providing corrective relational experiences. More specifically, evidence-based approaches can effectively reduce PTSD symptoms, but additional components are necessary to address DSO (i.e., affect dysregulation, negative self-concept, and relational problems), such as stabilization, interpersonal skills, and re-elaboration of relational patterns. Thus, the challenge lies in tailoring different approaches based on target and client profiles. Trauma-focused protocols, phase-based approaches, and adequate relational strategies appear to be more effective when delivered in a personalized, modular way (Cloitre et al., 2011; De Jongh and Hafkemeijer, 2024; Farina et al., 2025; Karatzias et al., 2019a,b).

### 4.2 Therapeutic factors: toward a process-based and relational account of change

A significant constraint in contemporary research is the over-reliance on symptom-reduction outcomes and diagnostic cutoffs in RCTs (Cloitre et al., 2011; Maercker et al., 2022). These methodologies frequently fail to capture substantial therapeutic change in domains such as identity, trust, and relational capacity (Levi, 2019; Pearlman and Courtois, 2005; Popolo et al., 2025). This demands a shift in perspective, moving from the focus on “what” changes in CPTSD—that is, the improvement of symptoms—to “how” change occurs, placing emphasis on the role of therapeutic processes and relational mechanisms (e.g., Laska et al., 2014). Indeed, effective treatment for CPTSD requires more than symptom-targeting treatments (Cloitre et al., 2011; Maercker et al., 2022). The realization of this objective requires sustained process-level change. In the treatment of CPTSD, the main therapeutic factors contributing to such a process-level change can be the following: the establishment of a therapeutic alliance, the development of trust, the repair of ruptures, the management of emotions, and the presence of a consistent therapeutic presence. These elements have been demonstrated to support safety and engagement while also creating conditions conducive to corrective emotional experiences and shifts in relational templates (e.g., Beaton and Thielking, 2019; Papa et al., 2024; Pearlman and Courtois, 2005; Popolo et al., 2025; see also Gelo and Podolan, 2025; Podolan and Gelo, 2023, 2024).

In the context of CPTSD, therapist-related factors, such as empathy, attunement, emotional responsiveness, and the capacity to maintain holding and containing functions, assume particular significance (Courtois, 2004; Levi, 2019; Matheson and Weightman, 2021; Su and Stone, 2020). These traits have been associated with positive outcomes in both structured and relational therapies. However, they are often not the focus of empirical research. In a similar manner, patient-related variables, including attachment organization, trauma history, readiness for change, emotional regulation capacities, and dissociative tendencies, have been demonstrated to influence the establishment of an alliance and the efficacy of therapy.

It is imperative to conceptualize the therapeutic alliance as a dynamic and active mechanism of change, rather than merely as a contextual or facilitative element (Wampold and Imel, 2015; see also Gelo et al., 2016). In long-term or psychodynamic therapies, for example, the alliance serves as the mechanism through which maladaptive relational patterns are reenacted and transformed. Something similar can be described also in other therapeutic approaches. These findings suggest that the quality of the alliance, rupture-repair cycles, and the therapist’s responsiveness to relational dynamics may explain significant variability in outcomes, particularly for symptoms associated with DSO (Matheson and Weightman, 2021; Papa et al., 2024; Popolo et al., 2025).

Nevertheless, these process variables are generally not incorporated into RCTs or meta-analyses, despite their established relevance. There is an imperative for process-outcome research and mechanism-focused study designs that evaluate the occurrence of change in therapy for CPTSD, as opposed to merely determining its presence. This includes the moment-to-moment tracking of alliance dynamics, the use of CPTSD-specific process measures, and the exploration of how dissociation or attachment shifts over the course of therapy.

It is essential to emphasize that these relational mechanisms should not be confined to psychodynamic or humanistic approaches. The integration of common relational factors—such as alliance-building, rupture repair, containment, and mentalization—into all therapeutic modalities is both feasible and necessary. This approach has the potential to enhance the efficacy of treatment, reduce treatment dropout rates, and improve long-term outcomes in CPTSD across a range of clinical contexts.

During the treatment process of CPTSD, challenges were found in forming therapeutic relationships, setting and maintaining boundaries and frames, focusing on relational and behavioral reenactments of past attachment, trauma, or loss, and handling dissociative processes, therapeutic ruptures, and dropout (Pearlman and Courtois, 2005). In CPTSD treatment, choosing the right approach and treatment plan depends on many factors: (1) patient’s symptom severity and current situation; (2) comorbidity with other psychiatric disorders; (3) trauma history and when the last trauma happened; (4) trauma characteristics such as onset, chronic, interpersonal, etc.; (5) medication; and (6) treatment history. Furthermore, as dissociation has been demonstrated to be associated with trauma symptoms (Hyland et al., 2019), it is essential that CPTSD treatment address dissociative experiences, as these experiences have been shown to impede an individual’s capacity to engage in therapy and maintain relationships, thereby hindering their ability to experience the present moment. However, a gap exists in the extant literature regarding the exploration of dissociation in the treatment of CPTSD, both in terms of outcomes and processes.

Overall, these findings suggest that the therapeutic factors of CPTSD can be ascribed to three clinically relevant, transtheoretical conceptual domains connected to DSO. The first domain is safety and therapeutic engagement. This domain includes trust and alliance building, setting appropriate boundaries, and therapeutic presence. This domain represents the minimum requirements for sustained treatment and reduced dropout risk. The second domain is affect regulation, which involves paying particular attention to dissociative experiences and pacing trauma-focused interventions. The third domain is relational learning and reorganization of the self through adequate management of rupture-repair cycles, responsive attunement, and corrective emotional experiences that impact existing relational patterns. Consistent with existing literature (Wampold and Imel, 2015), the therapeutic alliance is a dynamic change mechanism that enables clients to negotiate and transform their maladaptive relational patterns, with a positive impact on DSO (Pearlman and Courtois, 2005; Papa et al., 2024; Popolo et al., 2025; Courtois, 2004). In line with current transtheoretical approaches, these processes are not exclusive to a single therapeutic approach but can be conceptualized as common therapeutic factors that are operationalized differently in various approaches (Cloitre et al., 2004, 2011; Laska et al., 2014; Wampold and Imel, 2015). From this, it follows that such processes (e.g., alliance dynamics, rupture-repair cycles, and markers of emotional regulation and dissociation) should be assessed and monitored throughout treatment. These processes should also be associated with post-treatment outcomes, including DSO beyond PTSD symptoms.

### 4.3 Methodological problems

Despite the growing number of studies, the extant literature on CPTSD treatment is limited by several factors. First, there are problems with the *definition and assessment of outcomes*, especially with regard to the insufficient use of CPTSD-specific instruments and a lack of focus on DSO. This makes it difficult to compare findings from different studies. Second, there are *research design problems*, including small sample sizes, non-standardized outcome measures, and a lack of systematic comparisons among treatment approaches. Third, there is a *lack of process research*, with the majority of empirical research focusing on treatment outcomes. Consequently, our knowledge of the therapeutic factors of CPTSD treatment is limited. Case studies, which are frequent in the psychodynamic tradition and useful for describing treatment processes and stages, are informative but lack experimental control and generalizability (Spermon et al., 2010).

### 4.4 Implications for practice, training, and research

#### 4.4.1 Implications for practice

Clinicians should pay particular attention to personalized assessments and case formulations, especially concerning the distinction between PTSD core symptoms and CPTSD-specific DSO (Cloitre et al., 2011; Hyland et al., 2017, 2019). Furthermore, to maximize treatment effects, clinicians should prioritize a stabilization phase before trauma processing by fostering an adequate therapeutic setting, a safe relational environment and alliance building, modulating arousal, enhancing emotion regulation strategies, reducing dissociation, and favoring clients’ ability to stay in the present moment (Cloitre et al., 2011; Hyland et al., 2019; Papa et al., 2024; Pearlman and Courtois, 2005). This is consistent with the idea that failing to address emotional and relational instability adequately may limit the benefits of trauma processing (Bisson and Andrew, 2007; Cloitre et al., 2002; Cloitre et al., 2011; De Jongh and Hafkemeijer, 2024; Ford et al., 2005; Korn, 2009). Moreover, due to the maladaptive relational patterns of clients with CPTSD, clinicians should pay special attention to managing boundaries and termination (Beaton and Thielking, 2019; Herman, 1992b; Hyland et al., 2019; Korn, 2009; Pearlman and Courtois, 2005). Finally, clinical supervision may be extremely useful when the therapeutic relationship becomes stuck in reciprocal enactments due to a loss of the therapist’s intersubjective attunement (Salvatore et al., 2024).

#### 4.4.2 Implications for training

Practitioners should be trained to develop a “regulatory presence,” allowing them to recognize early signs of hyperactivation or hypoactivation. This enables them to foster clients’ grounding in the present moment and ensure interpersonal safety and continuity throughout the treatment process. Such an attitude transcends therapeutic approaches and is a prerequisite for any more directive or interpretive work (Courtois, 2004; Matheson and Weightman, 2021; Cloitre et al., 2011). Psychotherapy training should also provide practitioners with the ability to identify early rupture markers, provide relational repairs, and foster clients’ corrective emotional experiences (Papa et al., 2024; Popolo et al., 2025; Cloitre et al., 2004). Additionally, training should provide practitioners with the ability to steadily pace therapeutic work between stabilization and trauma processing, provide a predictable setting, and manage discontinuation and termination in order to reduce dropout risks (Pearlman and Courtois, 2005; Herman, 1992b; Beaton and Thielking, 2019). To this end, one fundamental aspect appears to be the practitioners’ ability to mentalize and attune intersubjectively. This ability should be adequately promoted in clinical supervision to allow practitioners to better understand what is happening in the therapeutic relationship and consequently orient their in-session work (Salvatore et al., 2024; Pearlman and Courtois, 2005).

#### 4.4.3 Implications for research

Regarding therapeutic outcomes, future studies should better and more systematically include DSO as dependent variables to increase the clinical validity of findings regarding the specificity of CPTSD and to make findings more comparable (Cloitre et al., 2011; Hyland et al., 2017; Hyland et al., 2019; Karatzias et al., 2019a,b). Research designs should be more robust and have greater statistical power by using bigger samples, adopting RCTs (especially for treatments with limited evidence, such as psychodynamic therapy), and making more systematic comparisons between conditions and treatment approaches. These designs should also systematically incorporate follow-up assessments to better understand the stability and maintenance of therapeutic outcomes (Bisson and Andrew, 2007; Cloitre et al., 2011; De Jongh and Hafkemeijer, 2024; Spermon et al., 2010; for a general overview, see Gelo et al., 2015). Additionally, these designs should account for how CPTSD treatment response differs based on types of developmental trauma exposure (e.g., domestic violence, emotional abuse/neglect) and comorbidity profiles (Cloitre, 2021; Hyland et al., 2021; Keyan et al., 2024). Regarding therapeutic factors, future process-focused and mixed-methods designs should more systematically assess different process variables longitudinally (e.g., alliance, rupture-repair, safety, dissociation, mentalizing, and therapist responsiveness) and determine whether they mediate CPTSD-specific outcomes (for a methodological account, see Gelo et al., 2012, 2020a,b). This should be done taking into account the complex and dynamic interaction of treatment process variables (for a methodological example, see Panayi et al., 2025; see also De Felice et al., 2019, 2020). In this way, it would be possible to provide a more accurate understanding of therapeutic factors in CPTSD treatment (Bateman et al., 2018; Cloitre et al., 2004, 2011; Hyland et al., 2019; Papa et al., 2024; Popolo et al., 2025).

### 4.5 Study limitations

The present study presents several limitations. First, the present paper is a narrative (non-systematic) review, which limits the reproducibility and generalizability of the findings. Future systematic reviews should adopt registered protocols for study searches, selections, and data extractions and syntheses. This would produce more generalizable findings and possibly cover other treatment approaches not addressed in this study. Second, the reviewed literature presents heterogeneous methodological constraints that may affect the reliability and validity of the discussed findings. Future systematic reviews should formally assess the risk of bias in the included studies and the quality of the body of evidence. Third, this paper focused only on individual psychotherapy, excluding other settings (e.g., group therapies and community-based approaches). Future studies should include different CPTSD treatment settings and formats. Finally, this paper focused on literature published in English. Future reviews should consider a broader range of languages.

## 5 Conclusion

The treatment of CPTSD requires a personalized yet flexible approach that addresses DSO beyond merely reducing CPTSD symptoms. To this end, therapists should work in a phase-sensitive manner, carefully oscillating between stabilization and trauma processing to promote an improvement of clients’ affect regulation, self-image, and relational functioning. In this context, these phases are not static sequences but rather flexible modules that therapists should use according to their clients’ individual resources and strategies. Existing approaches seem to share therapeutic processes mainly based on the therapist’s ability to intersubjectively attune with the client to provide an adequate, safe, relational environment that fosters learning processes and reorganization of the self, leading to a positive outcome. More systematic empirical research is needed to understand how psychotherapy for CPTSD works and to help clinicians and researchers develop more effective and appropriate treatments.
